# Electroencephalographic power spectrum changes in cerebral small vessel disease combined with cognitive dysfunction and its relationship with neutrophil/lymphocyte ratio and its clinical value – a pilot study

**DOI:** 10.3389/fneur.2023.1300240

**Published:** 2024-01-12

**Authors:** Xiaomin Guo, Zongwei Liu, Weishuai Yuan, Aiqin Wei, Guogang Luo

**Affiliations:** ^1^Department of Neurology, The First Affiliated Hospital of Xi’an Jiaotong University, Xi’an, China; ^2^Department of Neurology, The People’s Hospital of Shaanxi Province, Xi’an, China; ^3^First Institute of Oceanography, Ministry of Natural Resources, Qingdao, China

**Keywords:** cerebral small vessel disease, cognition impairment, neuropsychological assessment, quantitative electroencephalography, power spectrum analysis, neutrophil/lymphocyte ratio

## Abstract

**Objective:**

The study aimed to explore the changes in the electrical power spectrum of the brain and its correlation with neutrophil/lymphocyte ratio (NLR) in patients with cognitively impaired cerebral small vessel disease (CSVD) and to explore its clinical application.

**Methods:**

A total of 61 patients with CSVD who attended the People’s Hospital of Shaanxi Province from September 2021 to September 2022 were divided into the group with cognitive impairment (cerebral small vascular with cognitive impairment, CSVCI group, *n* = 29) and the group without cognitive impairment (CSVD group, *n* = 32) based on the Montreal Cognitive Assessment Scale (MoCA) score, while 20 healthy subjects were recruited as the control group (healthy control, HC group). EEG was performed in the three groups, and the difference in whole brain quantitative EEG power spectral density (PSD) was calculated and compared between the three groups.

**Results:**

The PSD values in the δ and θ bands of the CSVCI group were higher than those of the CSVD group, while the PSD values in the α band were lower than those of the CSVD and HC groups. In addition, PSD values in the δ-band in the CSVD group were lower than those in the HC group (all *p* < 0.05). Multifactorial logistic regression showed that reduced α-band global average PSD and low years of education were independent risk factors for cognitive impairment in patients with CSVD (*p* < 0.05). In patients with cerebral small-vessel disease, α-band PSD was positively and δ-band PSD negatively correlated with MoCA score, and paraventricular, deep white matter, and total Fazekas scores were negatively correlated with MoCA score. Furthermore, θ-band PSD is positively correlated with NLR (all *p* < 0.05).

**Conclusion:**

EEG activity was slowed down in patients with CSVD with cognitive impairment. The α-band global mean PSD values independently affected the occurrence of cognitive impairment in CSVD patients beyond the Fazekas score. NLR may be one of the mechanisms leading to the slowing down of the EEG, which can be used as an objective indicator for the early prediction of cognitive impairment but still needs to be clarified by further studies.

## 1 Introduction

Cerebral small-vessel disease (CSVD) is a general term for a series of clinicopathological syndromes that involve small blood vessels and capillary microcirculation in the brain, resulting in damage to the white matter and deep gray matter (1). CSVD has a wide range of etiologies and complex clinical manifestations, which may include focal neurological dysfunction, mood changes, subcortical dysfunction, and neurological dysfunction (2). In recent years, due to the impact of CSVD on cognitive function, neuropsychological assessment and biological and imaging markers have gradually become a hot research topic (3). However, more and more proofs have shown that structural imaging parameters lack sensitivity in early diagnosis of the disease, or structural phenotypes of the same disease entity are less consistent (4–6). Neuropsychological tests have a strong subjectivity and are affected by the patient’s mental state, education level, etc., and the diagnostic accuracy is not high enough. Biological tests such as blood and cerebrospinal fluid are invasive and lack specificity.

In contrast, electroencephalography (EEG), as a non-invasive method, is cheap, easily accessible, real time, and can be used to detect changes in cortical excitability, connectivity, and functional synchronization of EEG activity in patients with cognitive dysfunction, with good sensitivity and specificity for different types of cognitive dysfunction (7). In recent years, several research groups have used different analytical methods to find the relationship between EEG signals and Alzheimer’s disease (AD)/mild cognitive impairment (MCI), and the results have shown that the effects of AD/MCI on the EEG can be classified into the following categories (8): EEG activity slowdown, EEG complexity reduction, EEG synchronization perturbation, and gamma activity increase. Quantitative electroencephalography (QEEG) is a modern type of EEG analysis technique, and power spectral density (PSD) is the core part of QEEG, and PSD shows the strength of EEG signals and the frequency characteristics of EEG signals. However, the correlation between PSD and cognitive impairment in CSVD patients has not yet been reported in the literature.

In recent years, the study of inflammatory factors in CSVD has been gradually emphasized, especially the blood neutrophil-to-lymphocyte ratio has been found to be possibly an important risk factor for CSVD within the last 3 years (9–11) and may be associated with the occurrence of cognitive impairment in CSVD (12). However, whether there is a correlation between CSVD and EEG indicators remains unclear. In this study, we investigated the relationship between cognitive impairment and PSD and NLR in CSVD patients by EEG power spectrometry analysis in order to find early predictive biomarkers for the development of cognitive impairment in CSVD patients and to provide assistance in the clinical management of CSVD patients.

## 2 Materials and methods

### 2.1 Subjects

Thirty-two cases of CSVD patients, 21 male and 11 female subjects, with an average age of 63.91 ± 1.41 years, and 29 cases of CSVCI patients, 20 male and 9 female subjects, with an average age of 66.55 ± 1.37 years, who were hospitalized in Shaanxi Provincial People’s Hospital from September 2021 to September 2022, were enrolled in this study. On average, 20 cases of the same period of health checkups were collected as the control group, 8 male and 12 female subjects, with an average age of 61.30 ± 1.08 years. The inclusion criteria were as follows: 1. Age of 50–80 years old; 2. Examination in accordance with the diagnostic criteria of China’s 2021 edition of the Chinese Consensus on the Diagnosis and Treatment of Cerebral Small Vessel Disease (13); 3. Complete clinical data of patients or family members who are willing to join this study. The exclusion criteria were as follows: 1. Large cerebral infarction, cerebral hemorrhage, and other macrovascular lesions; 2. Encephalitis, normal pressure hydrocephalus, Alzheimer’s, Parkinson’s and other neurological diseases that cause cognitive dysfunction; 3. Other systemic diseases that cause cognitive changes, such as diabetes mellitus, obesity, and metabolic disorders; 4. People with color blindness and color weakness; 5. People with severe hearing, reading, and writing disorders; 6. Subjects with contraindications to MRI such as metal implants and claustrophobia. All subjects will be informed and sign a written informed consent form.

### 2.2 Clinical data acquisition

Basic data were collected from all patients, including age, sex, education, history of smoking, history of alcoholic consumption, history of hypertension, history of diabetes, MoCA scores, and scores of each cognitive domain during hospitalization. Hematological indices including total cholesterol (TC), triglyceride (TG), low-density lipoprotein-cholesterol (LDL-C), homocysteine (HCY), uric acid (UA) levels and blood neutrophil count (NE), lymphocyte count (Lym), and neutrophil/lymphocyte ratio (NLR) were collected for all subjects.

Cranial MRI data were acquired from a 3.0 T GE/Philips MRI scan. Periventricular white matter hyperintensity (PVH), deep white matter hyperintensity (DWMH), and total Fazekas scores were assessed by visual inspection.

Scalp EEG data were collected using a digital EEG detector (SOLAR2848B) from Beijing Sun Company, which utilized disk electrodes. Nineteen scalp disk electrodes (FP1, FP2, F3, Fz, F4, C3, Cz, C4, P3, Pz, P4, O1, O2, F7, F8, T3, T4, T5, and T6), grounding electrodes (Fpz), and bilaterally ear electrodes (A1, A2) were placed according to the international 10–20 standard system. The sampling frequency was set at 1000 Hz, and the impedance between the scalp and the electrodes was lower than 5 kΩ. The test was performed in a quiet, awake state. Subjects were required to cooperate with the instructions to complete the eyes open and closed test. After the EEG data acquisition was completed, the data were first pre-processed: using the eeglab toolbox in Matlab2021a software, including filtering (high-pass 0.5 Hz, low-pass 128 Hz, trapping 50 Hz and 100 Hz), removing the bad channels, ICA removing artifacts (ophthalmoelectricity, myoelectricity, electrocardiography, etc.), interpolating the bad channels, and averaging the reference for the whole brain. For the pre-processed EEG data, the pwelch function in Matlab2021a software was used to find the PSD of each EEG channel. In total, eight segments of data (10 s each) were selected for each channel under each event based on the principle of equal step length, and after calculating the PSD of each 10 s segment, the PSD of the eight segments was averaged, and the averaged PSD data were further divided into four frequency bands, including α-frequency band (8–14 Hz), β-frequency band (14–25 Hz), θ-frequency band (4–8 Hz), and δ-frequency band (0.5–4 Hz), and the PSDs of each frequency band were averaged as the feature data of quantized EEG.

### 2.3 Statistical analysis

Clinical data were analyzed using SPSS 25.0 software, and the measurement data were expressed as average ± standard deviation (^−^X ± S); the F test was used for comparison between groups, and the LSD method was used for two-by-two comparison. Categorical variable data, expressed as frequencies and percentages, were compared between groups using the chi-square test and two-by-two comparisons using the U-test. EEG data were pre-processed in Matlab2021a software using the eeglab toolbox for EEG signals. The pwelch function was used in Matlab2021a software to find the power spectral density (PSD) of each EEG channel. EEG data were analyzed in Matlab2021a software using the self-contained ttest2 function (two-by-two comparisons of the feature data of the quantified EEGs were performed using the independent samples *t*-test). Pearson’s correlation was used to analyze the correlation between the global average PSD and MoCA scores under four frequency bands in the three groups of patients. Multivariate binary logistic regression analysis was used to identify risk factors for the development of cognitive impairment in patients with CSVD. Differences were considered statistically significant at a *p*-value of <0.05.

## 3 Results

### 3.1 Clinical characteristics

There were differences between the three groups in terms of age, years of education, and history of hypertension. Furthermore, two-by-two comparisons between groups revealed that age was higher in the CSVCI group than in the HC group (*p* = 0.013), years of education were lower in both the CSVCI group than in both the CSVD and HC groups (*p* < 0.001), and both the CSVCI and CSVD groups were higher than the HC group in terms of percentage of hypertensive disease (*p* < 0.001). The rest of the general information did not differ between the three groups.

Hematologic indicators did not show significant differences among the three groups.

The results of the comparison of imaging indexes showed that the DWMH and the total Fazekas scores were higher in the CSVCI group than in the CSVD group and the HC group, and they were also higher in the CSVD group than in the HC group (all *p* < 0.001). In addition, the PVH Fazekas score was higher in the CSVCI group and the CSVD group than in the HC group (both *p* < 0.001, Table 1).

**Table 1:** Comparison of baseline information among the three groups.

	HC group (*n* = 20)	CSVD group (*n* = 32)	CSVCI group (*n* = 29)	*F*/*χ*^2^	*p*
Age (years)	61.30 ± 1.08	63.91 ± 1.41	66.55 ± 1.37^a^	3.28	0.043
Male (*n*, %)	8 (40.00)	21 (65.00)	20 (69.00)	4.74	0.094
Educational years (years)	11.25 ± 0.79	11.03 ± 0.57	7.66 ± 1.37^ab^	8.87	<0.001
History of alcoholic consumption (*n*, %)	4 (20.00)	9 (28.10)	8 (27.60)	0.49	0.784
History of smoke (*n*, %)	4 (20.00)	11 (34.40)	11 (37.90)	1.87	0.392
History of hypertension (*n*, %)	3 (15.00)^b^	27 (84.40)^a^	25 (86.20)^a^	36.49	<0.001
History of diabetes (*n*, %)	2 (10.00)	6 (18.80)	7 (24.10)	3.54	0.473
TC (mmol/L)	4.41 ± 0.28	3.89 ± 0.16	3.77 ± 0.16	2.77	0.069
TG (mmol/L)	1.71 ± 1.45	1.46 ± 0.79	1.36 ± 0.79	0.80	0.452
LDL-C (mmol/L)	2.46 ± 0.19	2.15 ± 0.11	2.01 ± 0.12	2.66	0.076
HCY (umol/L)	13.01 ± 5.15	17.73 ± 12.83	17.67 ± 8.72	1.67	0.195
UA (umol/L)	307.85 ± 122.11	323.40 ± 99.48	298.47 ± 73.44	0.51	0.604
Fazekas (PVH)	0.35 ± 0.49^b^	1.34 ± 0.75^a^	1.86 ± 1.03^ab^	20.75	<0.001
Fazekas (DWMH)	0.50 ± 0.61	1.53 ± 0.57^a^	1.86 ± 1.06^a^	18.48	<0.001
Fazekas (total)	0.85 ± 1.04^b^	2.88 ± 1.24^a^	3.72 ± 2.05^ab^	20.95	<0.001
NE (*10^9/L)	1.70 ± 0.45	1.79 ± 0.58	1.59 ± 0.50	0.62	0.542
Lym (*10^9/L)	3.16 ± 1.36	3.61 ± 1.44	3.47 ± 1.50	1.18	0.312
NLR	1.98 ± 0.94	2.11 ± 0.65	2.42 ± 1.37	1.28	0.284

TC, total cholesterol; TG, triglyceride; LDL-C, low-density lipoprotein-cholesterol; HCY, homocysteine; UA, uric acid; PVH, periventricular white matter hyperintensity; DWMH, deep white matter hyperintensity; NE, neutrophil; Lym, lymphocyte; NLR, neutrophil/lymphocyte ratio.

a A statistically significant between-group comparison with the HC group.

b A statistically significant between-group comparison with the CSVD group.

### 3.2 Comparison of PSD in the three groups

**Figure 1:**
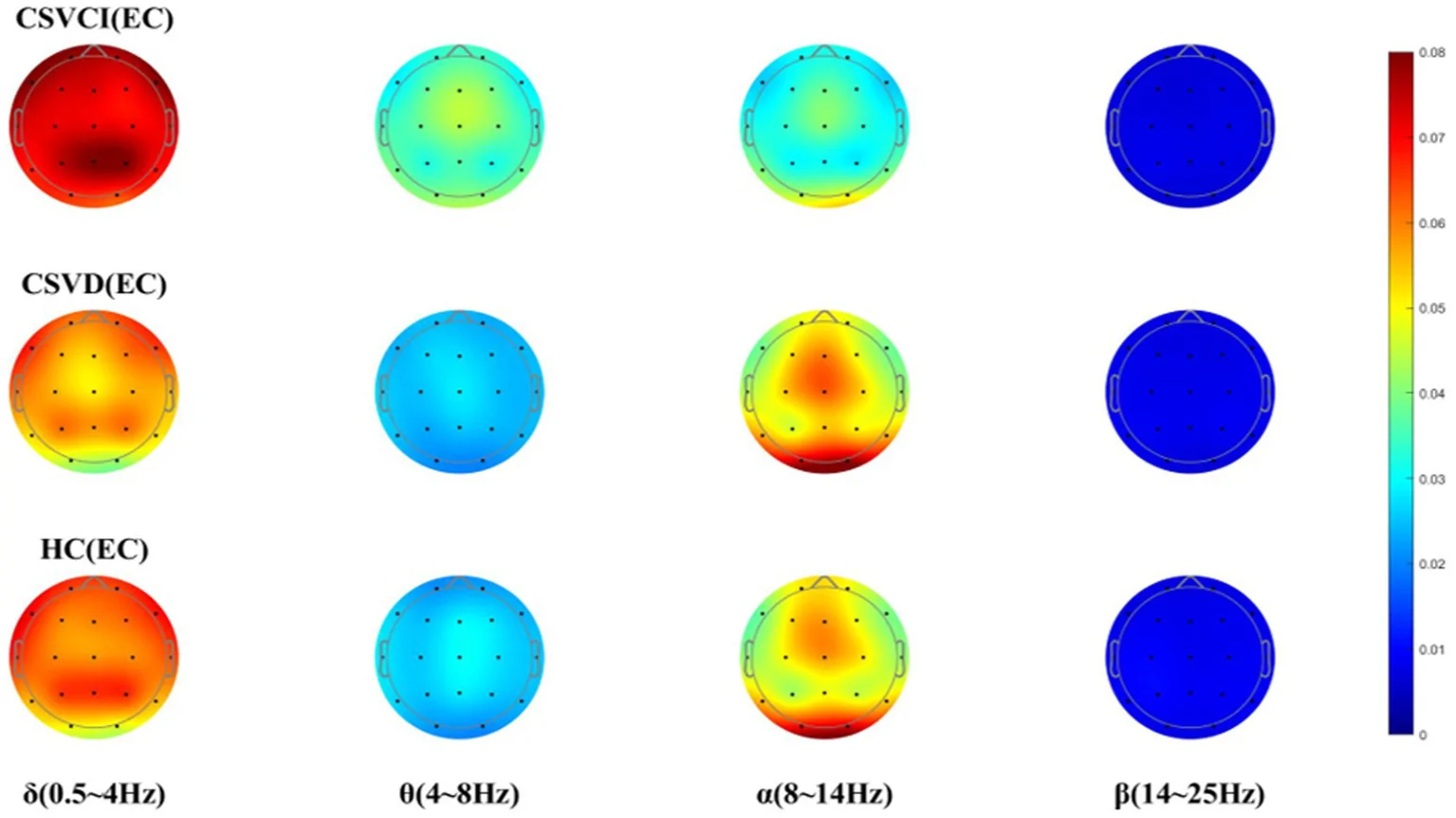
Topography of whole brain power in different frequency bands in three groups of patients. It can be visualized that the theta-band power spectrum of the CSVCI group increased, while the alpha-band power spectrum decreased compared with the other two groups.

**Figure 2:**
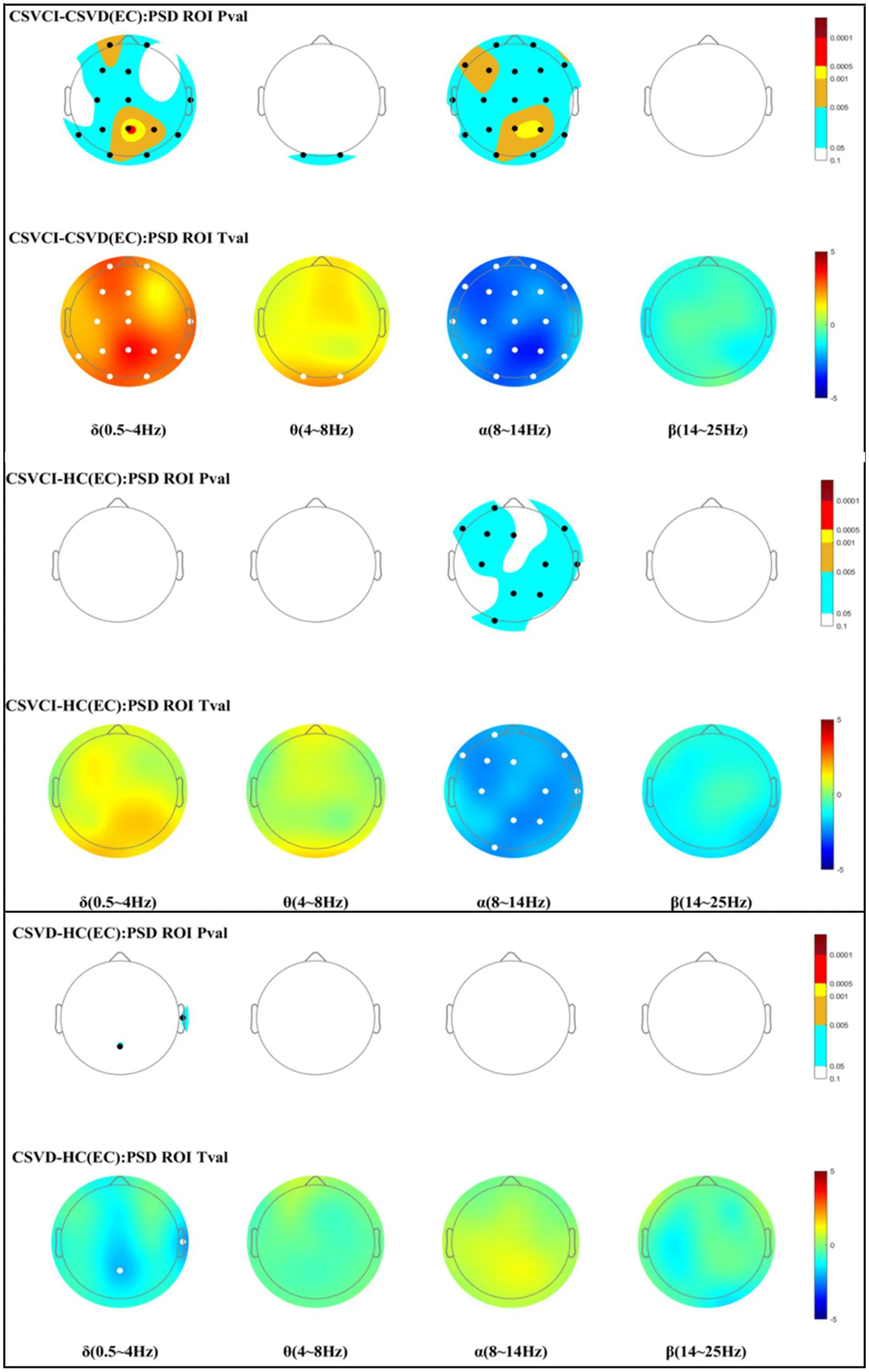
*p*-value and *T*-value statistical graphs for one-by-one comparisons of PSD between the three groups. For *p*-value statistical graphs, for PSD under different frequency bands, channels with significant differences between two groups are marked with black dots, while different colors are used to represent the degree of significant differences, with the redder color representing the more significant difference. For *T*-value statistical graph, for PSD under different frequency bands, the channels with significant differences between the two groups are marked with white dots, the blue color in the graph represents that the *T*-value is negative, which means that the front group is smaller than the back group, and the darker the blue color, it means that the difference is more significant, the red color in the graph represents that the *T*-value is positive, which means that the front group is larger than the back group, and the darker the red color, it means that the difference is more significant.

There were differences in global PSD values in the α, δ, and θ frequency bands, but not in the β frequency band, between the three groups. Comparing the two groups, the PSD values in the δ-band in the CSVCI group were higher than those in the CSVD group in FP1, FP2, F3, Fz, C3, Cz, P3, Pz, P4, O1, O2, T5, and T6 channels. The PSD values in the θ-band in the CSVCI group were higher than those in the CSVD group in the O1 and O2 channels. The PSD values in the α-band in the CSVCI group were lower than those in the CSVD group in almost all brain regions except the T4 channel and were lower than those in the HC group in FP1, FP2, F3, Fz, F8, C3, C4, Pz, P4, and O1 channels. The PSD values of the δ-band in the CSVD group were lower than those in the HC group in T4 and Pz channels (all *p* < 0.05, Figures 1, 2).

### 3.3 Multivariate logistic regression analysis

Patients with CSVD with or without cognitive impairment were used as the dependent variable, and indicators that differed between groups in the *t*-test and α- and δ-frequency bands of global average PSD were included as independent variables, and the results suggested that years of education and the PSD value in α-band were negatively associated with the occurrence of cognitive impairment in CSVD patients (all *p* < 0.05, Table 2).

**Table 2:** Multivariate binary logistic regression analysis of factors affecting cognitive impairment in CSVD patients.

	*β*	SE	*p*	OR	95% CI
Age	0.040	0.051	0.434	1.041	0.941–1.151
Sex	−1.631	0.989	0.099	0.196	0.028–1.360
History of hypertension	−1.865	1.198	0.121	0.156	0.015–1.637
Educational years	−0.382	0.130	0.003*	0.683	0.529–0.880
α-band PSD	−66.427	29.287	0.023*	1.353 × 10^−29^	1.622 × 10^−54^–1.130 × 10^−5^
δ-band PSD	20.248	28.831	0.428	16.219*10^8^	1.790 × 10^−16^–2.160 × 10^33^
Fazekas (PVH)	1.205	0.905	0.183	3.336	0.567–19.649
Fazekas (DWMH)	−0.457	0.921	0.619	0.633	0.104–3.845
NLR	0.184	0.361	0.611	1.201	0.592–2.438

*p < 0.05. PVH, periventricular white matter hyperintensity; DWMH, deep white matter hyperintensity; NLR: neutrophil/lymphocyte ratio.

### 3.4 Correlation analysis

**Figure 3:**
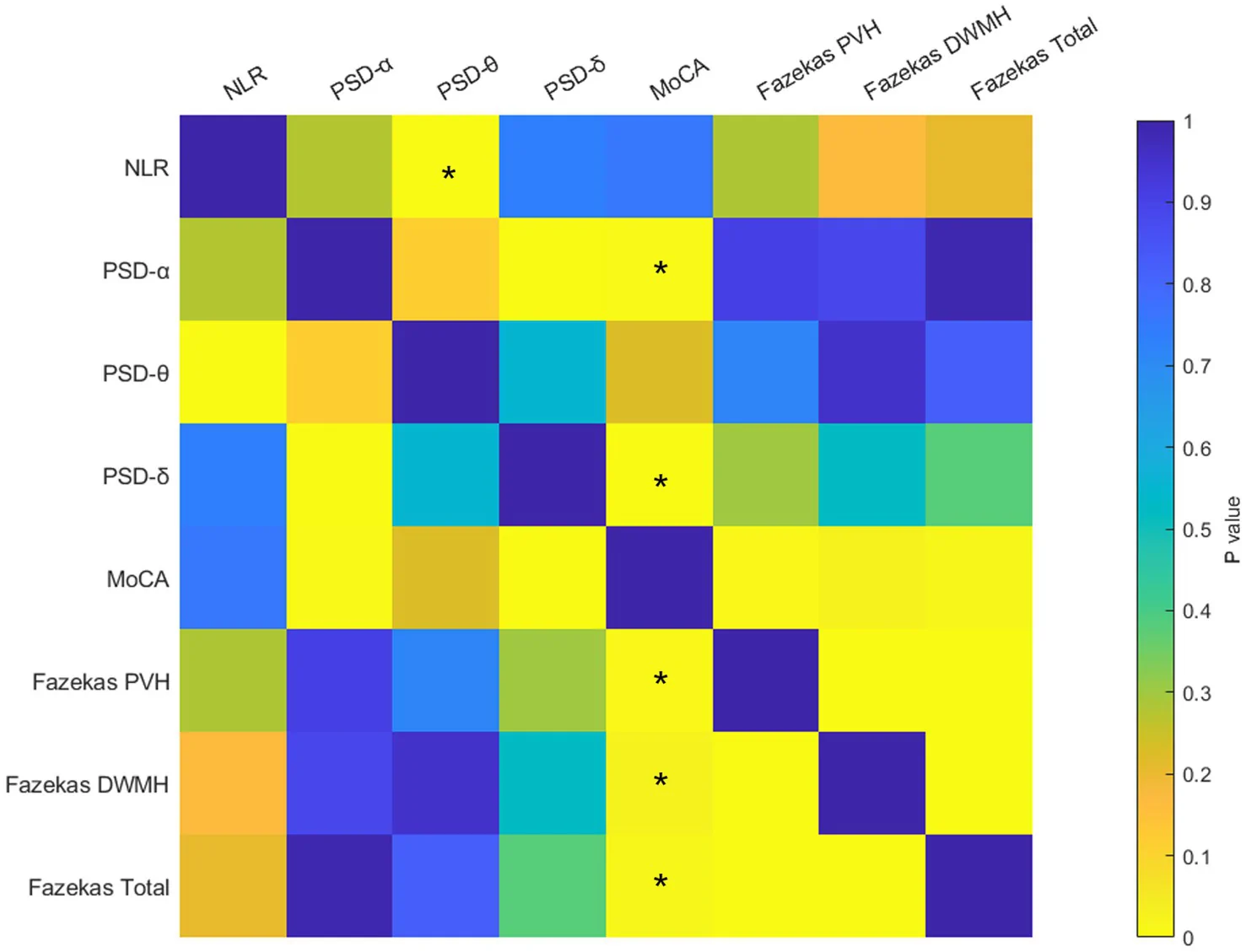
Correlation matrix of differential indices. MoCA was seen to be positively correlated with global α band PSD, while negatively correlated with global δ band PSD, PVH Fazekas, DWMH Fazekas, and total Fazekas score. In addition, NLR was positively correlated with global θ band PSD. *p < 0.05.

**Figure 4:**
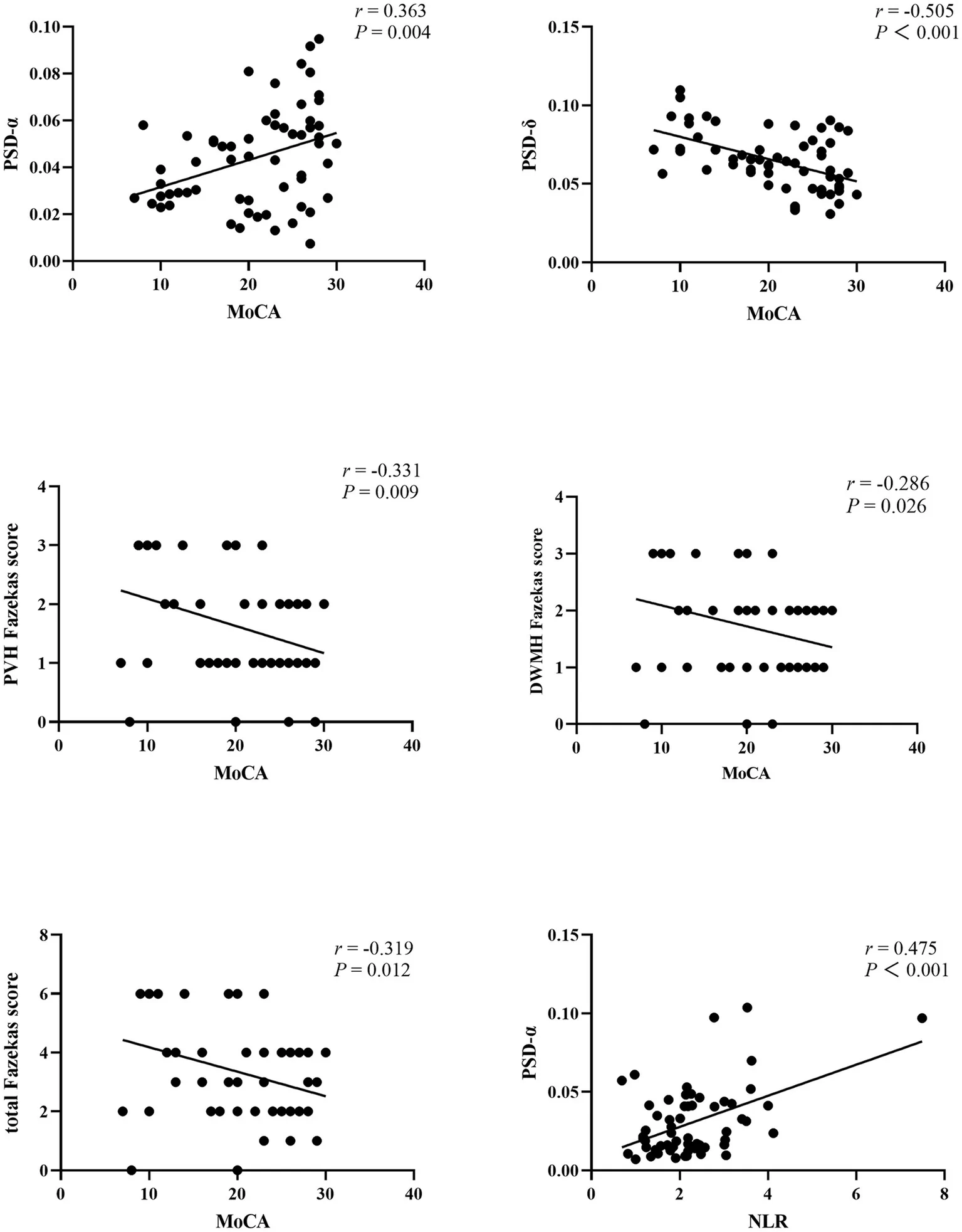
Correlation scatterplot for indicators with positive correlation.

Pearson’s correlation analysis of α- and δ-band global average PSD values, Fazekas scores, and MoCA scores in patients with CSVD and healthy controls, respectively, revealed that in patients with CSVD, the α-band PSD values were positively correlated with the MoCA score (*p* = 0.004, *R* = 0.363), whereas the δ-band PSD values were negatively correlated with the MoCA score (*p* < 0.001, *R* = −0.505). In addition, the PVH Fazekas score (*p* = 0.009, *R* = −0.331), the DWMH Fazekas score (*p* = 0.026, *R* = −0.286), and the total Fazekas score (*p* = 0.012, *R* = −0.319) were negatively correlated with MoCA score. While NLR was significantly and positively correlated with θ-band whole-brain mean PSD (*p* < 0.001, *R* = 0.475) (Figures 3, 4).

## 4 Discussion

The aim of this study was to investigate the PSD changes in CSVD patients who developed cognitive dysfunction and its relationship with NLR. The results of the study showed that the PSD values of CSVCI patients were mainly characterized by an increase in δ-activity and a decrease in α-activity in almost all brain regions, suggesting a slowing down of electroencephalographic activity in patients with small-vessel disease accompanied by cognitive dysfunction. The results of multivariate logistic regression indicated that decreased global average PSD values in the α-band were a risk factor for the development of cognitive impairment in patients with cerebral small-vessel disease independently of the severity of cerebral white matter hyperintensities. Correlation analysis showed that MoCA scores in CSVD patients were positively correlated with PSD in the α-band and negatively correlated with PSD in the δ-band and also positively correlated with the severity of cerebral white matter hyperintensities, whereas there was no such correlation in healthy controls, suggesting that the PSD values in the relevant bands may be a potentially objective indicator to assist clinical diagnosis and treatment. In addition, correlation analysis also revealed a positive correlation between whole-brain mean theta-band PSD and NLR in patients with cerebral small-vessel disease, suggesting that NLR may lead to slowing down of EEG activity and cause or exacerbate cognitive deficits through inflammatory mechanisms.

An increase in the slow frequency bands (δ and θ) and decrease in the amplitude and power of the fast frequency bands (α and β) have also been found in many previous studies on AD patients and patients with mild cognitive impairment (14, 15). The results of the present study are consistent with this. The mechanism by which CSVD leads to cognitive dysfunction has not yet been fully elucidated, and it is hypothesized that it may be a combination of various risk factors and genetic factors that lead to brain dysfunction. Srinivasan et al. (16) concluded that EEG can reflect the physiological changes and functional alterations of the cerebral cortex and can directly reflect the cerebral cortex function. Physiologically, EEG cortical activity depends on a complex balance between different neurotransmitter systems, mainly cholinergic pathways. Alpha rhythms are mainly regulated by thalamocortical interactions that facilitate or inhibit the transmission of sensory-motor and cognitive information between the subcortical and cortical pathways (17). Therefore, researchers have hypothesized that the reduced amplitude of cortical rhythms in patients with CSVCI may be related to the impairment of the cholinergic pathway, which results in the abnormal increase in cortical excitation or disinhibition in the resting state. In addition, Musaeus et al. found a positive correlation between temporal area δ-band power and total cerebrospinal fluid tau protein in patients with AD, suggesting that the increase in slow waves may be associated with abnormal deposition of tau protein (15). The increased δ-band PSD in CSVCI patients found in this study may then suggest that cognitive impairment occurs in CSVD patients due to abnormal deposition of tau protein for some reason. This hypothesis is supported by the study of Kim et al. who found that both CSVD scores and β-amyloid were independently associated with tau protein in patients with subcortical vascular cognitive impairment (18).

In order to clarify the independent effect of the EEG indicators on cognitive impairment in CSVD patients, imaging indicators were co-incorporated into the analysis in this study. The findings showed that Fazekas scores were higher in the CSVCI group and that Fazekas scores were negatively correlated with MoCA scores in patients with CSVD, suggesting that the severity of CSVD itself affects patients’ cognitive function. This has also been reported in a previous study (19). However, it is worth noting that although the F-test results showed that the Fazekas scores differed among the three groups, the results of multivariate logistic regression analyses that included them with EEG and general clinical indicators as independent variables showed that only the α-band global average PSD was negatively correlated with the occurrence of cognitive deficits, whereas there was no significant correlation with the Fazekas scores, which suggests that the α-band average PSD has a negative influence on the occurrence of cognitive deficits in CSVD patients. This suggests that the effect of α-band global average PSD on the development of cognitive deficits in CSVD patients is independent of white matter damage, i.e., brain function changes may aggravate cognitive deficits in CSVD patients independently from white matter structural damage, which means that the EEG power spectrum may be mediated by factors other than anatomical damage that are independent of those visible on imaging to cause cognitive dysfunction. However, it must be recognized that this point still needs to be clarified by further studies. The mechanism may be related to the abnormal deposition of tau protein described above.

Rodriguez et al. reported that MMSE scores were negatively correlated with relative power at 2.0–6.0 Hz and positively correlated with bilateral hemispheric relative power at 6.5–12.0 Hz (20). This is generally consistent with the results of the present study, suggesting that increased PSD in the δ-band and decreased PSD in the α-band may be a clinical marker of early neurological decline. In addition, a study applying transcranial magnetic stimulation (TMS) to intervene in AD patients showed that there was a mild increase in α-frequency band power in the whole brain of AD patients after 6 months of intervention and that α-band power was positively correlated with MMSE scores after intervention (21), suggesting that neuromodulation therapy may be used as a new therapeutic tool for AD and that α-band power may serve as an objective predictor of efficacy. Similarly, the present study found that the correlation between α- and β-band PSD values and MoCA scores in patients with CSVD also suggests that they may be used as an objective indicator to evaluate the cognitive severity of patients with CSVD and to predict the efficacy of neuromodulation interventions. In particular, the global average PSD values in the α-band may serve as an objective indicator for early prediction of cognitive impairment in CSVD patients. However, this still needs to be validated by further large-sample, randomized controlled intervention studies.

It is important to note that the main result of the present study is the difference between the CSVCI group and the other two groups, which suggests that the slowing down of the EEG activity in the resting state mainly represents the onset of cognitive impairment but not CSVD itself. In a study applying EEG power spectroscopy to assess AD, high-risk ApoE gene carriers without dementia and healthy controls (22), it was found that for EEG in the resting, closed-eye state, there were differences mainly in AD patients, whereas for EEG in the hyperventilation state, there were differences mainly in high-risk ApoE gene carriers without dementia (or pre-AD), suggesting that EEG spectrum power in different states may represent different periods of cognitive impairment. In contrast, the EEG analyzed in the present study were resting, closed-eye state EEG and thus may be a more accurate characterization of cognitive impairment. Despite the small number of results, the present study also found differences in the PSD between the CSVD and HC groups, but it is worth noting that the CSVD group had lower EEG slow-wave activity than the HC group, which may represent compensatory brain activity in the pre-cognitive phase.

Several previous studies have confirmed that NLR is an important risk factor for CSVD (9–11) and may be involved in the mechanism by which it develops cognitive impairment (12). In recent years, some studies have also reported that elevated NLR in the acute phase may be a predictor of cognitive dysfunction after stroke (23, 24). Although the present study did not directly find a relationship between NLR and cognitive impairment in CSVD, the results of correlation analysis showed that NLR was significantly correlated with theta-band PSD in CSVD patients, which laterally suggests that NLR may lead to electroencephalogram slowing in CSVD patients through inflammatory mechanisms and further lead to or aggravate cognitive impairment. This suggests that early detection of NLR and timely anti-inflammatory intervention in CSVD patients may improve their cognitive prognosis, but further studies are needed to clarify this.

In summary, the results of the present study suggest that the slowing of EEG activity in CSVD may be an early marker for the development of cognitive deficits, and its mechanism may be related to tau protein deposition and that PSD values in the α- and δ-bands in patients with CSVD may be used as an objective indicator for assessing clinical severity and predicting the efficacy of interventional treatments, and in particular, the global average PSD value in the α-band may be used as an early predictor of the development of cognitive deficits in patients with CSVD independently of from white matter hyperintensities. However, the results of this study must be interpreted with the following limitations in mind: first, this study is a single-center, small-sample, cross-sectional pilot study, and the generalizability of the results needs to be further confirmed by a large-sample, multicenter, prospective observational study. Second, the EEG analysis method applied in this study is a basic power spectrum analysis, which can only reflect the average intensity of brain function activity during a certain period of time locally and cannot accurately reflect the functional connection between different brain regions and the dynamic changes of brain function at different times. Further studies can apply wavelet conversion, non-linear EEG analysis methods, complexity entropy, and other methods to comprehensively characterize the changes in brain function. Finally, the incomplete imaging data led to the study not take the total burden of CSVD as a covariate for corrective analysis, and the results of the study may be affected by the different degrees of severity of other lesions in CSVD except for the white matter hyperintensity, and further studies can combine the multimodal neuroimaging methods to explore its brain function changes in more depth, to improve the sensitivity and specificity of the diagnosis of CSVCI, and to provide assistance for the early detection and treatment of CSVCI.

## Data availability statement

The original contributions presented in the study are included in the article/supplementary material, further inquiries can be directed to the corresponding author.

## Ethics statement

The studies involving humans were approved by Shaanxi Province People’s Hospital Ethics Committee. The studies were conducted in accordance with the local legislation and institutional requirements. The participants provided their written informed consent to participate in this study.

## Author contributions

XG: Conceptualization, Writing – review & editing. ZL: Formal analysis, Methodology, Writing – review & editing. WY: Formal analysis, Methodology, Writing – review & editing. AW: Data curation, Formal analysis, Writing – original draft. GL: Investigation, Writing – review & editing.
