# Correction: Electroencephalographic power spectrum changes in cerebral small vessel disease combined with cognitive dysfunction and its relationship with neutrophil/lymphocyte ratio and its clinical value—A pilot study

**DOI:** 10.3389/fneur.2026.1945317

**Published:** 2026-09-15

**Authors:** Xiaomin Guo, Zongwei Liu, Weishuai Yuan, Aiqin Wei, Guogang Luo

**Affiliations:** 1 Department of Neurology, The First Affiliated Hospital of Xi'an Jiaotong University, Xi'an, China; 2 Department of Neurology, The People's Hospital of Shaanxi Province, The Third Affiliated Hospital of Xi'an Jiaotong University, Xi'an, China; 3 First Institute of Oceanography, Ministry of Natural Resources, Qingdao, China

**Keywords:** cerebral small vessel disease, cognition impairment, neuropsychological assessment, quantitative electroencephalography, power spectrum analysis, neutrophil/lymphocyte ratio

In the published article, the affiliation ‘Department of Neurology, The First Affiliated Hospital of Xi'an Jiaotong University, Xi'an, China' was erroneously omitted for author Xiaomin Guo. This affiliation has now been added for author Xiaomin Guo. The order of affiliations has also been corrected: affiliation 3 is now affiliation 1, affiliation 1 is now affiliation 2, and affiliation 2 is now affiliation 3.

The original version of this article has been updated.

